# Multiple cerebral sinus thromboses complicating meningococcal meningitis: a pediatric case report

**DOI:** 10.1186/1471-2431-14-147

**Published:** 2014-06-13

**Authors:** Elena Bozzola, Mauro Bozzola, Giovanna Stefania Colafati, Valeria Calcaterra, Annachiara Vittucci, Matteo Luciani, Alberto Villani

**Affiliations:** 1 Department of Pediatrics, Pediatric and Infectious Diseases Unit, Bambino Gesù Children’s Hospital, IRCCS, Rome, Italy; 2 Fondazione IRCCS San Matteo Pavia, Department of Internal Medicine, University of Pavia, Pavia, Italy; 3 Neuroradiology Unit, Department of Imaging, Bambino Gesù Children’s Hospital, IRCCS, Rome, Italy; 4 Department of Hematology, Oncology and Trasfusion Medicine, Bambino Gesù Children’s Hospital, IRCCS, Rome, Italy

**Keywords:** Cerebral venous sinus thrombosis, Meningitis, Children, Thrombophilic evaluation

## Abstract

**Background:**

Cerebral venous sinus thrombosis (CVT) is a rare and potentially life-threatening condition in the pediatric population. The clinical presentation is frequently nonspecific; thus diagnosis is often delayed or missed.

**Case presentation:**

A previously healthy 8 month-old boy was diagnosed with meningococcal meningitis. At hospital admission, an urgent non contrast-enhanced computed tomography (CT) of the head and neck was performed with normal results. Ceftriaxone was promptly started and the clinical condition of the patient improved. However, on the 7^th^ day of hospitalization, the child suddenly manifested irritability and lethargy. An urgent contrast-enhanced CT of the head and neck was immediately performed, revealing thrombosis of the superior sagittal, transverse and rectus sinuses. A thrombophilic evaluation was performed, revealing hyperhomocysteinemia and methylenetetrahydrofolate reductase (MTHFR) variants (C677T and A1298C).

**Conclusions:**

The causes of CVT may be categorized into three main groups: hypercoagulable states, conditions causing blood flow disturbances, and all causes of inflammation or infection. In this case report, we observed more than one risk factor that predisposed the patient to CVT. Consequently, even if a causative factor is detected, a thrombophilic blood evaluation should be performed. In fact, in case of a prothrombotic condition, the patient’s family should be advised that prompt administration of anticoagulant is necessary in the event of situations that could lead to thrombosis. Finally, CVT may be considered a possible complication of infection even when recent imaging results are normal. A prompt CVT diagnosis is required to obtain a good outcome. Delayed diagnosis is mainly due to the rarity of the disease and physicians’ unawareness of this type of complication.

## Background

Cerebral venous sinus thrombosis (CVT) is a rare, potentially life-threatening disorder that may have vital or morbid consequences, such as motor dysfunction or visual impairment. It predominantly affects adults in their third and fourth decades [1]. Even if high-quality epidemiologic studies are lacking, the available data suggest an incidence of 0.67 cases per 100,000 children per year. The prevalence of childhood CVT varies according to age; neonates are the most commonly affected [2-4].

The signs and symptoms of CVT are frequently nonspecific, often resulting in delayed or missed diagnosis [5]. In fact, CVT may present with a variety of signs and symptoms, including irritability, headache, seizure, encephalopathy, papilledema, cranial nerve palsies, motor weakness and coma [6]. Studies suggest that prompt diagnosis and anticoagulant therapy may improve outcome in the majority of patients [2,4,7]. The causes of CVT may be categorized into three main groups according to Virchow’s classification. The first group includes all hypercoagulable states. The second group consists of conditions causing blood flow disturbances. Finally, the third group comprises all causes of inflammation or infection [8]. Among these, prothrombotic conditions, either genetic or acquired, malignancy, and head injury are the most frequent causes. As regards infectious causes of CVT, these are responsible for only 6 to 12 percent of cases [9]. Previous reports have cited sinusitis, otitis, and mastoiditis among the principal predisposing factors. Although the frequency of septic thrombosis is decreasing due to antibiotic development, recent studies have demonstrated that CVT may be a complication of meningitis and sepsis in about 7% and 16% of cases, respectively [2,7,10].

We describe the clinical case of a child with multiple sinovenous thromboses occurring after the diagnosis of meningococcal meningitis.

## Case presentation

**Figure 1:**
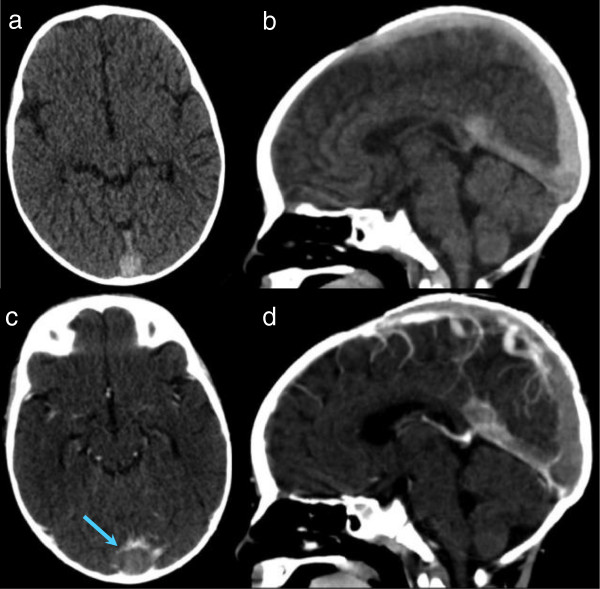
**Head computed tomography images without (a,b) and with (c,d) contrast; axial (a,c) and reformatted sagittal (b,d) images.** Note the empty delta sign (c, arrow).

**Figure 2:**
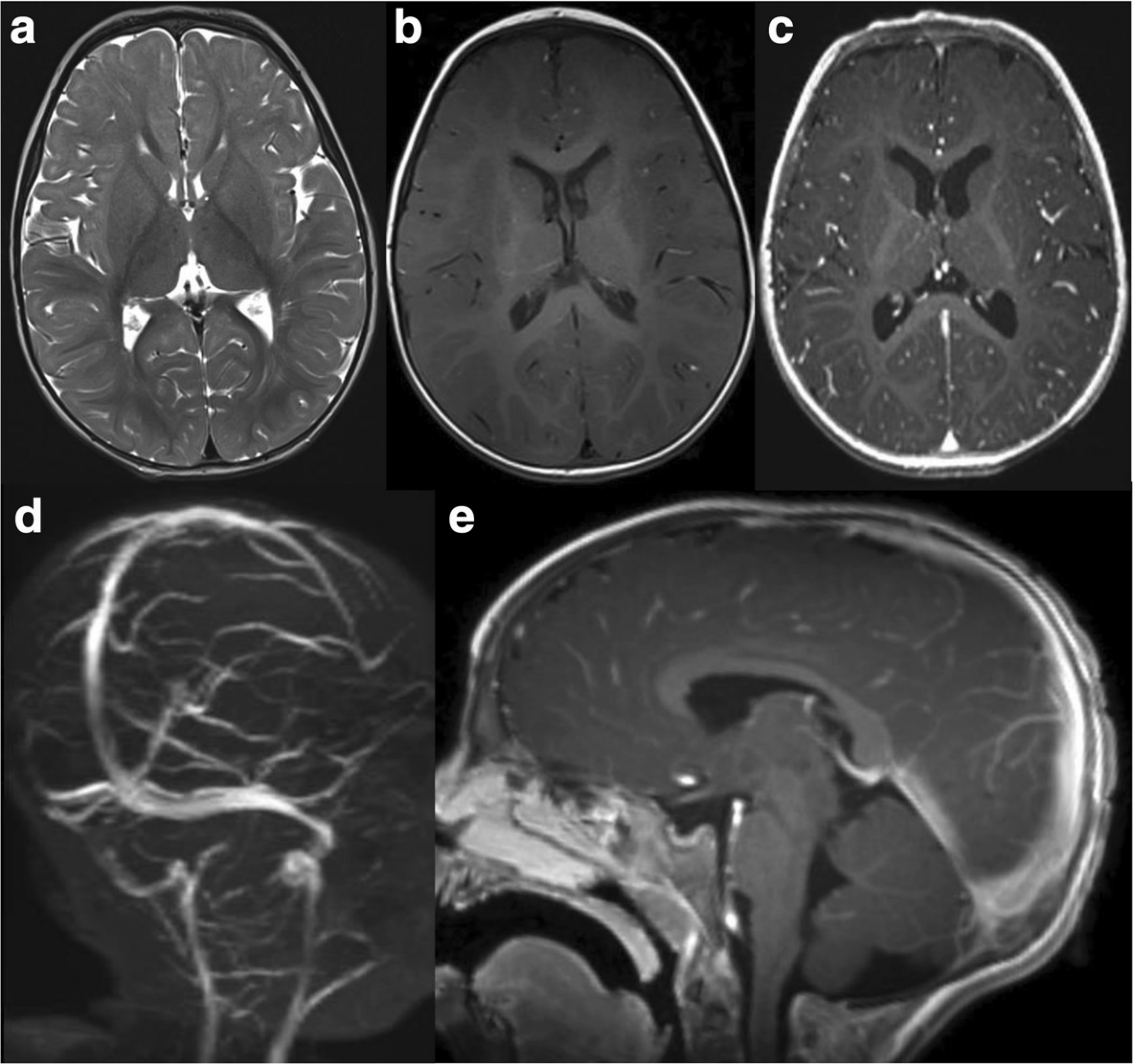
Axial T2w (a) and axial T1w images without (b) and with contrast (c), magnetic resonance venography (d) and sagittal T1w with contrast (e).

A previously healthy 8 month-old boy presented at the Emergency Department with high-grade fever, vomiting, and macular erythema of both upper limbs. No signs or symptoms of ear and upper respiratory infection were reported. Physical examination revealed a febrile, obtunded, and hypotonic child. Otoscopic findings as well as throat examination were normal. Neck stiffness was noted. No neurological deficit or papilledema was present. Laboratory tests showed leukocytosis (27000/μL; normal value > 6000 and <17000/μL) and an increased C-reactive protein (19,19 mg/dl; normal value below 0,5 mg/dl). An urgent non-contrast computed tomography (CT) of the head and neck was performed, with normal results. Bacterial meningitis was suspected and a lumbar puncture confirmed the diagnosis. The cerebrospinal fluid (CSF) was opalescent with 33900 white blood cells, increased total protein (304,2 mg/dl) and normal glucose levels (37 mg/dl). Serum glucose level was also normal (106 mg/dl).The child received intravenous dexamethasone and ceftriaxone as empiric treatment. Neisseria Meningitis C was confirmed in both blood and CSF cultures. Two days later, the child’s condition improved, with fever resolution. Suddenly, seven days after hospital admission, the child manifested irritability and lethargy. Moreover, a bulging fontanel was noted at the physical examination. An urgent contrast-enhanced CT of the head and neck was immediately performed and revealed thrombosis of the superior sagittal, transverse and rectus sinuses (Figure 1). Heparin treatment was started in order to recanalize the occluded sinuses and to prevent the propagation of thrombi. A thrombophilic evaluation was performed which revealed hyperhomocysteinemia and methylenetetrahydrofolate reductase (MTHFR) variants (C677T and A1298C). One week later, a new CT revealed a recanalization of the previously involved sinuses, without parenchymal damage. Consequently, the patient was discharged under treatment with subcutaneous low molecular weight heparin (LMWH). At follow up five months later, the child was in good clinical condition with normal magnetic resonance imaging (MRI) results (Figure 2). The anticoagulant therapy was discontinued, but the family was advised to administer anticoagulants in case of situations that could predispose to thrombosis.

## Conclusions

According to the literature, CVT may be caused by hypercoagulable states, by conditions causing blood flow disturbances, or by inflammation/infection [5]. In our case report, we observed more than one risk factor that predisposed to CVT. We speculate that the genetic prothrombotic condition predisposed to CVT, which was triggered by a central nervous system infection. In fact, the CT scan was normal at both meningitis onset and post-discharge follow-up. For this reason, thrombophilic evaluation should be encouraged in all children with CVT, independent of other established risk factors such as meningitis, in order to identify children with increased risk factors for thrombophilia. Thrombophilia may be defined as a high risk for thrombosis, which can be either inherited or acquired. The percentage of patients with CVT who have an identified risk factor for thrombophilia varies significantly from study to study, from 33% to 99% [11]. Moreover, there is no consensus as to which baseline studies should be included in a standard hypercoagulability evaluation [12]. In one report, anticardiolipin antibody was the most common prothrombotic risk factor, while in another the homozygous MTHFR mutation was the most common [2,8]. A German study reported cases in which either elevated lipoprotein A or protein C deficiency was associated with CVT; in an American study, protein C and antithrombin III deficiencies were common [7,13]. Other potential procoagulant markers recently considered in CVT include anemia, hyperhomocysteinemia, and factor VIII levels.

In the event of a prothrombotic diagnosis, the patient’s family should be advised to administer drugs (antiplatelet or anticoagulant agents) in situations that could lead to thrombosis. In fact, patients recovering from CVT are at risk of recurrence or other thrombotic event [8]. It should be explained to patients that testing of their first degree relatives for heritable thrombophilia will allow the detection of affected but asymptomatic family members.

Because of its nonspecific presentation, diagnosis of CVT may be delayed and even missed. Clinicians must maintain a high index of suspicion when treating any child with an acute central nervous system infection and presenting neurological signs and symptoms despite administration of appropriate therapy.

Imaging should be considered to rule out intracranial complications. The currently available diagnostic tools include head CT, MRI and MR venogram [4,6]. Of these, head CT is the most commonly performed examination when sinus thrombosis is suspected [2]. Diagnostic sensitivity is increased with the addition of a postcontrast CT. The empty delta sign, a classic signature of CVT, is present on post-contrast CT in 10% to 30% of cases. On head CT with contrast, the empty delta sign may be seen as a triangular pattern of contrast enhancement surrounding a central region which lacks contrast enhancement in the posterior part of the superior sagittal sinus [2,4]. A dense cord sign (hyperdense fresh blood clot) is seen on noncontrast head CT in 1% to 5% of cases [4]. A previous normal result, even if performed just a few days prior, should not influence the clinician’s decision to repeat the exam. In case of CVT, follow-up imaging of the venous sinuses is generally performed in order to check thrombosis evolution. The mainstay of CVT treatment is anticoagulation and supportive care. The purpose of anticoagulant therapy is to limit the propagation of an existing clot and to prevent the formation of further thrombi [2,4]. Previous studies have reported encouraging data on the safety of anticoagulation therapy in children with CVT and on improved cognitive outcome [2,7]. Although definitive evidence of effectiveness is lacking, there is a general consensus that anticoagulation with unfractionated intravenous heparin or subcutaneous LMWH is appropriate treatment for acute CVT [14]. The treatment most used for infants and children is LMWH for 7–10 days (120-180 UI/kg twice a day in children up to 1 year of age, and 100 UI/Kg twice a day in older patients) followed by oral anticoagulants or antiplatelets generally for 3–6 months [7]. Therapy is generally discontinued 6 months after the acute phase but may be prolonged after hematological evaluation depending on the patient’s age and clinical condition. Instructions to the family at patient’s discharge include the necessity to continue anticoagulation after the acute phase in order to prevent CVT recurrence.

Finally, CVT in children sometimes has a poor prognosis despite prompt diagnosis and therapy. Commonly identified morbidities include motor dysfunction, neuropsychiatric disorders, visual impairment, pseudotumor cerebri, seizures, and headaches [4]. In our case, even though CVT involved multiple sinuses and the child was affected by both meningitis and sepsis, the prognosis was good, with no sequelae at five months follow-up. The early diagnosis led to prompt therapy and consequently enabled a good neurodevelopmental outcome.

## Consent

Written informed consent was obtained from the patient’s mother for publication of these data and for the accompanying images. A copy of the written consent is available for review by the Editor of this Journal.

## Competing interests

The authors have no competing interests to declare.

## Authors’ contribution

BE provided medical assistance to the patient and collected medical information, BM and CV revised the literature, CGS supervised the neuroradiological examination included in the case report, LM supervised the hematological examination included in the case report, VA was involved in the clinical follow-up of the patient, VA supervised the patient treatment plan. All authors read and approved the final manuscript.

## Pre-publication history

The pre-publication history for this paper can be accessed here:

http://www.biomedcentral.com/1471-2431/14/147/prepub

## References

[B1] Algahtani HA Aldarmahi AA Cerebral sinus venus thrombosis Neurosciences (Riyadh) 2014 19 1 11 16 24419443

[B2] deVeber G Andrew M Adams C Bjornson B Booth F Buckley DJ Camfield CS David M Humphreys P Langevin P MacDonald EA Gillett J Meaney B Shevell M Sinclair DB Yager J Canadian Pediatric Ischemic Stroke Study Group Cerebral sinovenous thrombosis in children N Engl J Med 2001 345 6 417 423 10.1056/NEJM200108093450604 11496852

[B3] Saposnik G Barinagarrementeria F Brown RD Jr Bushnell CD Cucchiara B Cushman M deVeber G Ferro JM Tsai FY American Heart Association Stroke Council and the Council on Epidemiology and Prevention Diagnosis and management of cerebral venous thrombosis: a statement for healthcare professionals from the American Heart Association/American Stroke Association Stroke 2011 42 1158 10.1161/STR.0b013e31820a8364 21293023

[B4] Carpenter J Tsuchida T Cerebral sinovenous thrombosis in children Curr Neurol Neurosci Rep 2007 7 2 139 146 10.1007/s11910-007-0009-3 17324365

[B5] Matar NE Rassi SJ Melkane AE Haddad AC Lateral sinus thrombosis in the pediatric population: multiple presentations for a potentially lethal disease Pediatr Emerg Care 2009 25 10 681 683 10.1097/PEC.0b013e3181bec90c 19834419

[B6] Hedlund GL Cerebral sinovenous thrombosis in pediatric practice Pediatr Radiol 2013 43 2 173 188 10.1007/s00247-012-2486-z 23212594

[B7] Sébire G Tabarki B Saunders DE Leroy I Liesner R Saint-Martin C Husson B Williams AN Wade A Kirkham FJ Cerebral venous sinus thrombosis in children: risk factors, presentation, diagnosis and outcome Brain 2005 128 477 489 10.1093/brain/awh412 15699061

[B8] Ferro JM Canhão P Stam J Bousser MG Barinagarrementeria F ISCVT Investigators. Prognosis of cerebral vein and dural sinus thrombosis: results of the International Study on Cerebral Vein and Dural Sinus Thrombosis (ISCVT) Stroke 2004 35 664 10.1161/01.STR.0000117571.76197.26 14976332

[B9] Carvalho KS Bodensteiner JB Connolly PJ Garg BP Cerebral venous thrombosis in children J Child Neurol 2000 16 574 580 11510928 10.1177/088307380101600807

[B10] Fitzgerald KC Golomb MR Neonatal Arterial Ischemic Stroke and Sinovenous Thrombosis Associated With Meningitis J Child Neurol 2007 22 818 10.1177/0883073807304200 17715272

[B11] Chan A de Veber G Prothrombotic disorders and ischemic stroke in children Semin Pediatr Neurol 2000 7 301 308 10.1053/spen.2000.20075 11205719

[B12] Mullins M Grant P Wang B Gonzales RG Schaefer PW Parenchymal abnormalities associated with cerebral venous sinus thrombosis: assessment with diffusion-weighted MR imaging Am J Neuroradiol 2004 25 1666 1675 15569728 PMC8148707

[B13] Heller C Heinecke A Junker R Knofler R Kosch A Kurnik K Schobess R von Eckardstein A Strater R Zieger B Nowak-Gottl U Childhood Stroke Study Group Cerebral venous thrombosis in children: a multifactorial origin Circulation 2003 108 1362 1367 10.1161/01.CIR.0000087598.05977.45 12939214

[B14] Coutinho JM Stam J How to treat cerebral venous and sinus thrombosis J Thromb Haemost 2010 8 877 20149074 10.1111/j.1538-7836.2010.03799.x

